# Evolving public views on the value of one’s DNA and expectations for genomic database governance: Results from a national survey

**DOI:** 10.1371/journal.pone.0229044

**Published:** 2020-03-11

**Authors:** Forrest Briscoe, Ifeoma Ajunwa, Allison Gaddis, Jennifer McCormick

**Affiliations:** 1 Smeal College of Business, The Pennsylvania State University, University Park, Pennsylvania, United States of America; 2 Industrial and Labor Relations (ILR) School, Cornell University, Ithaca, New York, United States of America; 3 Schreyer Honors College, The Pennsylvania State University, University Park, Pennsylvania, United States of America; 4 Department of Humanities, College of Medicine, Penn State Clinical and Translational Science Institute, Penn State University Hershey, Hershey, Pennsylvania, United States of America; University of Brescia, ITALY

## Abstract

We report results from a large survey of public attitudes regarding genomic database governance. Prior surveys focused on the context of academic-sponsored biobanks, framing data provision as altruistic donation; our survey is designed to reflect four growing trends: genomic databases are found across many sectors; they are used for more than academic biomedical research; their value is reflected in corporate transactions; and additional related privacy risks are coming to light. To examine how attitudes may evolve in response to these trends, we provided survey respondents with information from mainstream media coverage of them. We then found only 11.7% of respondents willing to altruistically donate their data, versus 50.6% willing to provide data if financially compensated, and 37.8% unwilling to provide data regardless of compensation. Because providing one’s genomic data is sometimes bundled with receipt of a personalized genomic report, we also asked respondents what price they would be willing to pay for a personalized report. Subtracting that response value from one’s expected compensation for providing data (if any) yields a net expected payment. For the altruistic donors, median net expected payment was -$75 (i.e. they expected to pay $75 for the bundle). For respondents wanting compensation for their data, however, median net expected payment was +$95 (i.e. they expected to receive $95). When asked about different genomic database governance policies, most respondents preferred options that allowed them more control over their data. In particular, they favored policies restricting data sharing or reuse unless permission is specifically granted by the individual. Policy preferences were also relatively consistent regardless of the sector in which the genomic database was located. Together these findings offer a forward-looking window on individual preferences that can be useful for institutions of all types as they develop governance approaches in this area of large-scale data sharing.

## Introduction

The use of human genomic data collections is expanding, fueled by declining technological costs and enthusiasm for the promise of precision medicine [1–2]. Accordingly, various organizations responsible for managing enormous genomic biobanks are developing and refining their governance systems–i.e., the organizational structures and policies that shape data collection, data integrity, data end uses, transparency, stakeholder input processes, and data security–seeking to balance the benefits of broad data use with the need to mitigate risk and meet societal responsibilities [3–4].

Public expectations regarding the collection and use of genetic data are important and influential variables for the design of such governance systems. Prior survey research in this area has usually focused on the limited context of research biobanks owned by academic institutions, and has emphasized the notion that the individuals providing their genetic data are acting as altruistic biospecimen donors [5–10]. Respondents have been asked how they feel about their data being shared with researchers, whether their data should be retained for unspecified future research uses, and how their privacy should be protected. Findings in this context suggest that most participants, within the sole context of non-profit research biobanks, are generally willing to donate their data, are comfortable with indefinite use of their data, and are reassured by moderate privacy protections.

Yet the context of previous research presents an incomplete profile of public expectations for genetic database governance. We note that governance expectations for genetic databases in the future will be informed by two developing social phenomena: growing awareness of both the commercial value of genomic data and the emerging privacy risks for individuals providing data. Recent media coverage of deals such as Glasko Smith Kline’s partnership with 23andme and Roche’s purchase of Flatiron Health extend public knowledge of genomic data’s commercial value. And awareness of privacy and security risks may be rising as a result of news reports about police use of genomic databases [11–13], and of data breaches involving genomic, health and other sensitive personal data [14–16]. Because genomic databases rely on public participation, it is important to ask how these recent social phenomena could influence public expectations.

As an attempt to develop a more robust understanding of public expectations, the Genomic Data Governance Survey (GDGS) assessed individuals’ willingness to contribute genomic data, and also their views on genomic governance policies. The respondents were U.S.-based, and were first provided with publicly-available information from news reports on how genomic databases are being used, commercial transactions involving these data, and potential benefits as well as risks for individuals providing their data. After learning this information, respondents were asked about their willingness to provide their data, any payment they expect to receive in exchange for doing so, and how different database governance policies would alter their willingness to provide data.

## Materials and methods

### Survey design and administration

The GDGS was fielded from November 27 to December 20, 2018. The survey questionnaire was developed based on in-depth field interviews with officials and employees involved in genomic governance at 12 different organizations. Those field interviews were conducted between January 2017 and September 2018. Once the questionnaire was created, it was then pretested in a pilot survey administered to 174 undergraduate students using the Qualtrics online platform in Pennsylvania State University’s Smeal Behavioral Research Lab in October 2018.

After pre-testing and subsequent updates to the survey instrument, the nationally-representative GDGS survey was then administered by Qualtrics, using their Panels recruitment service and online platform. For population-representative surveys such as this, Qualtrics utilizes pre-assembled panels. Surveys are administered using a serial router that employs weighted randomization in order to assign surveys to respondents. Upon entry into the router, panelists are first checked to ensure they have not exceeded survey participation limits. Once matched to the survey based on profile information, panelists are asked screening questions within the router to meet project criteria (detailed below). Qualtrics also employs a standard routine (which uses attention checks) to remove responses from speeders and straight-liners.

To further ensure national representativeness on key demographic characteristics, Qualtrics employed screening questions to capture respondent age, sex, race/ethnicity, education level, and employment status. This ensured U.S. population representativeness based on five key dimensions: gender (final sample 49.7% female); race (61.9% non-Hispanic White, 17.3% Hispanic, 12.3% Non-Hispanic Black, 5.3% Asian, 3.2% Other Race); age (median 46, range 18–90); employment status (59.7% employed), and educational attainment (12.9% Less than high school diploma, 27.6% High school or equivalent, 20.8% Some college but not degree, 8.2% Associate degree, 19.0% Bachelor’s degree, 8.3% Master’s degree, 3.3% Professional degree or doctorate).

Following the practice of other recent surveys on public attitudes toward genomic data [6,10], respondents were provided with information about genomic databases using a short (3 minute) recorded video. This video was introduced after the completion of the initial screening questions, but prior to the start of the main survey (transcript text provided in S1 Appendix). The video was intended to provide respondents with concise, factual information with minimal audio-visual distraction. It was also intended to focus on the commercial value of personal information including genomic sequence data, to begin to answer the question of how growing awareness of the commercial value of genomic data, and emerging privacy risks for individuals providing data, might impact both expectations for governance as well as willingness to contribute to any biobank or genomic sequence repository.

To reduce inattention bias, respondents who failed at least one of 3 simple comprehension check questions immediately following the presentation of the informational video were excluded from the final sample. In addition, respondents who failed an additional attention check included near the end of the survey were also eliminated from the final sample. Additional methods used by Qualtrics to ensure high quality responses for this survey include a speeding screener and an initial quality commitment question. For respondents included in the final sample, completion time averaged 18.2 minutes, inclusive of the time required to watch the informational primer.

Excluding those screened out to ensure demographic representativeness, and comprehension/speeder exclusions, a total of 3515 individuals entered the survey, of whom 2020 completed it, for an effective survey completion rate of 57.5%.

### Questions and response categories

Respondents were randomly assigned to a specific organization type in order to evaluate willingness to provide genomic data. The five possible organization types, with corresponding one-sentence descriptions provided to respondents, were: “Genetic Data Inc. is a U.S. for-profit technology corporation”; “GreatCare Hospital is a U.S. non-profit hospital system”; “Genomics & Health Research Lab is located at Middle State University”; “BioPharmaCo is a global for-profit drug company”; and “The National Institutes of Health (NIH) is a U.S. federal research agency.” We consciously used the term hospital rather than academic medical center. As noted other surveys examining similar issues have framed the biobanking collection as taking place at an academic medical center or medical center closely affiliated with a university.

To gauge baseline willingness to provide genomic data, respondents were then told about an opportunity coming from the organization to which they were randomly assigned. The vignette text was identical, except for organization names and descriptions. For example, the for-profit vignette text read: “Now imagine that today you are presented with an opportunity to provide your DNA data to Genetic Data Inc., to be used in a biomedical research program. They can obtain your DNA data if you provide them with a small saliva sample. Genetic Data Inc. is a U.S. for-profit technology corporation. Based on this information, and what you have just learned about DNA data, how willing would you be to provide your DNA data to Genetic Data Inc.? Choose one.” The four unordered response options were: “Willing as a charitable donation” (referred to as ‘donors’); “Willing if I'm paid at least a certain amount of money” (referred to as ‘sellers’); “Unwilling, at least for now” and “Unwilling, now or ever” (jointly referred to as ‘unwilling’).

‘Seller’ respondents (i.e. those who answered the baseline willingness question using “Willing if I’m paid at least a certain amount of money”) were then asked a follow-up question regarding how much payment they expected in order to provide their genomic data. The question referenced the same organization they were previously assigned. For example: “About how much would you expect to be paid by Genetic Data Inc. for them to keep your DNA data in their database? Please enter an amount in U.S. dollars (but do not include the "$" sign).” Respondents answered the question by entering a monetary value into an open-ended response box. Text entries were restricted to exclude negative values, values larger than 1,000,000, and non-numeric responses. If a restricted value was entered, the respondent was prompted to use a numeric value between $0 and $1,000,000.

To capture the financial value respondents placed on obtaining a personal genomic report, all respondents were also asked, “Now imagine that you have an opportunity to purchase a report that will provide you with information about your ancestry, and forecast your risk for 20 different genetically based health conditions. To obtain this report, the seller needs a saliva sample with your DNA in it. However, in this case, your DNA data will not be stored or used for any other purpose. How much would you be willing to pay for this report?” Respondents answered using the same open-ended response box with the same text restrictions described above.

All respondents were also asked to react to a series of different genomic data governance policies. Twelve policies were presented to all respondents, in a randomized order to reduce potential question order bias. After each policy, respondents were asked to choose between five response options (“reduce willingness greatly,” “reduce willingness somewhat,” “no effect,” “increase willingness somewhat,” and “increase willingness greatly”). The 12 policy statements are listed in S2 Appendix.

### Data analysis

The data were imported into STATA (version 16) for analysis. Only completed survey responses were included in the final analysis dataset. No additional data-cleaning or response-exclusion criteria were applied at the analysis stage. For statistical significance tests reported, we used a p-value cut off of 0.05. STATA code and data files are available upon request.

For analysis and reporting purposes, we focused on comparing three groups as defined by responses to the baseline willingness question described above (‘donor,’ ‘seller,’ and ‘unwilling’ respondents).

To analyze and compare payment expectations, we used all ‘seller’ responses to the payment question, and assumed that all ‘donor’ respondents were willing to provide their genome without any expected payment (i.e. payment = $0). ‘Unwilling’ respondents were not included in the payment analysis. However, a follow-up question asking ‘unwilling’ respondents how much payment they would need to overcome their unwillingness yielded higher average values than ‘seller’ respondents.

To calculate payment expectations for a bundled transaction, we used the following procedure. For each ‘seller’ respondent, we subtracted their personal genomic report price from their expected payment amount, to generate that respondent’s net implied payment for a bundled transaction. For each ‘donor’ respondent, we subtracted their report price from an expected payment of $0, to generate that respondent’s net implied payment for a bundled transaction. Implied payments for the bundled transaction were then compared across ‘seller’ and ‘donor’ groups.

To assess differences across groups in governance policy responses (which are ordinal variables), we employed two-group Wilcoxon rank-sum (Mann-Whitney) tests and multi-group Kruskal-Wallis equality-of-populations rank tests. Frequency tables on which these tests were calculated are provided in S3 Appendix. To test for differences in the frequency of ‘donor’, ‘seller’, and ‘unwilling’ response categories across organization types, we used a chi-squared test for independence. Differences in sellers’ mean compensation expectations across organization types were assessed using mean equality tests (assuming homogeneity).

## Results

### Public willingness to provide genomic data and conditions for exchange of data

After being provided with the information in the 3-minute video described above, only 11.7% (n = 234) of respondents were willing to provide their data as an altruistic donation (‘donor’ respondents). Fully 50.6% (n = 1022) of respondents were willing to provide it if compensated with a payment of some amount (‘seller’ respondents), and another 37.8% (n = 764) were unwilling to provide it even if payment was available (‘unwilling’ respondents). These results contrast with previous surveys that focused on the context of academic research biobanks, which report rates of willingness consistently over 50% [5–6,10].

We also assessed the dollar amounts that those identified as sellers were seeking in exchange for their data. Asked in an open-ended format, the median reported value among sellers was $130 (mirroring the amount paid per genome in a recent commercial transaction that had been summarized in our priming information). This finding suggests that the pre-survey priming video did influence perceptions and responses, as anticipated, thus reflecting what could happen as individuals encounter real-life information alerting them to the valuation of genetic data. Though few comparisons are available, this finding also appears broadly consistent with a 2008 study in which a hypothetical $50 payment yielded willingness to participate in a genomic research biobank among 52% of respondents [5].

**Fig 1:**
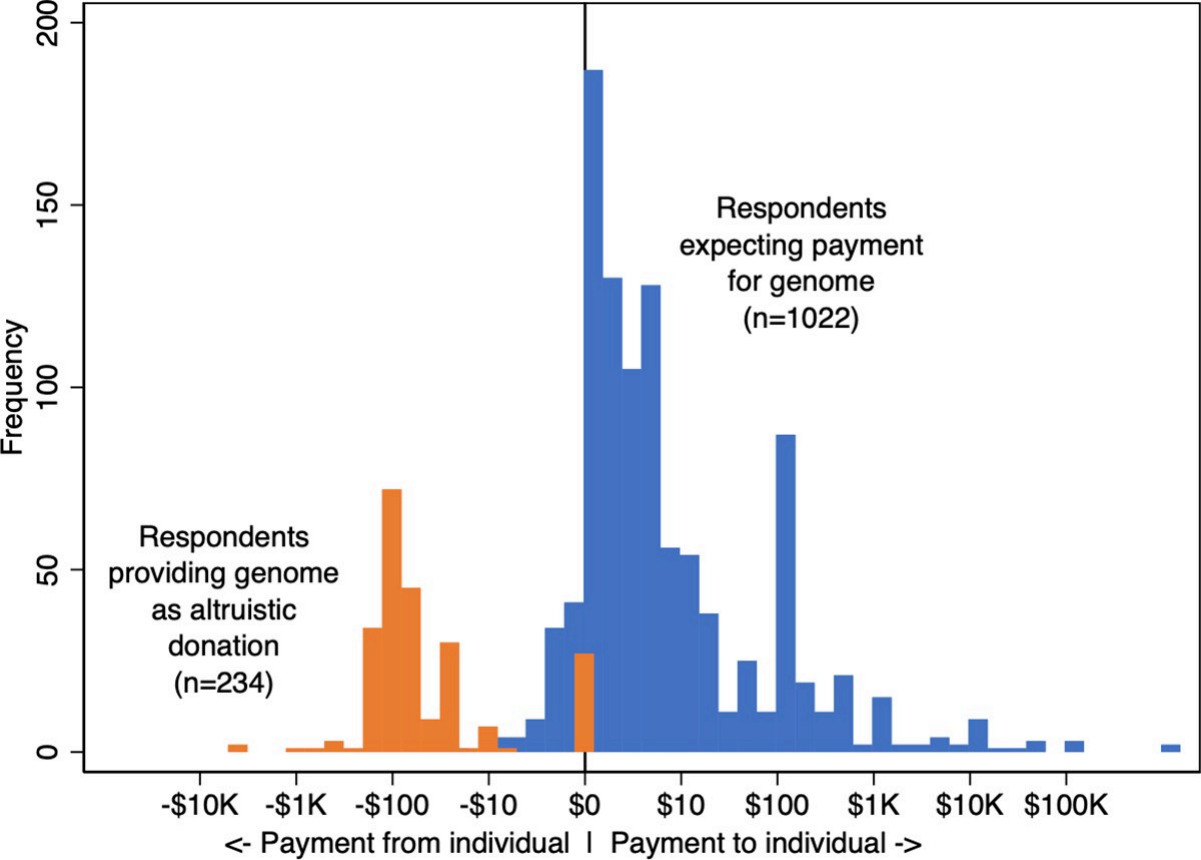
Net implied payment for a bundled transaction in which individual provides genomic data and receives personal report.

Provision of genomic data is sometimes bundled with receipt of a personalized genomic report. This bundled-transaction format is used by direct-to-consumer (DTC) genomics companies (e.g. donation includes a payment to the company), and will be used by the NIH’s *All of Us* program (no payment required). Therefore, we also asked respondents about the price they would be willing to pay for a personalized genomic report, so that we could subtract that from their expected payment (if any) to yield a net expected payment for the bundled transaction. Results are displayed in Fig 1. For sellers, the median implied value of a bundled transaction was $95 paid to them. In contrast, for respondents identified as donors, the median implied value was $75 paid by them. The deal that donors expect is somewhat less than a typical DTC offering; however, donors only represented a small minority of respondents in our survey.

### Effect of database governance policies on willingness to provide genomic data

**Fig 2:**
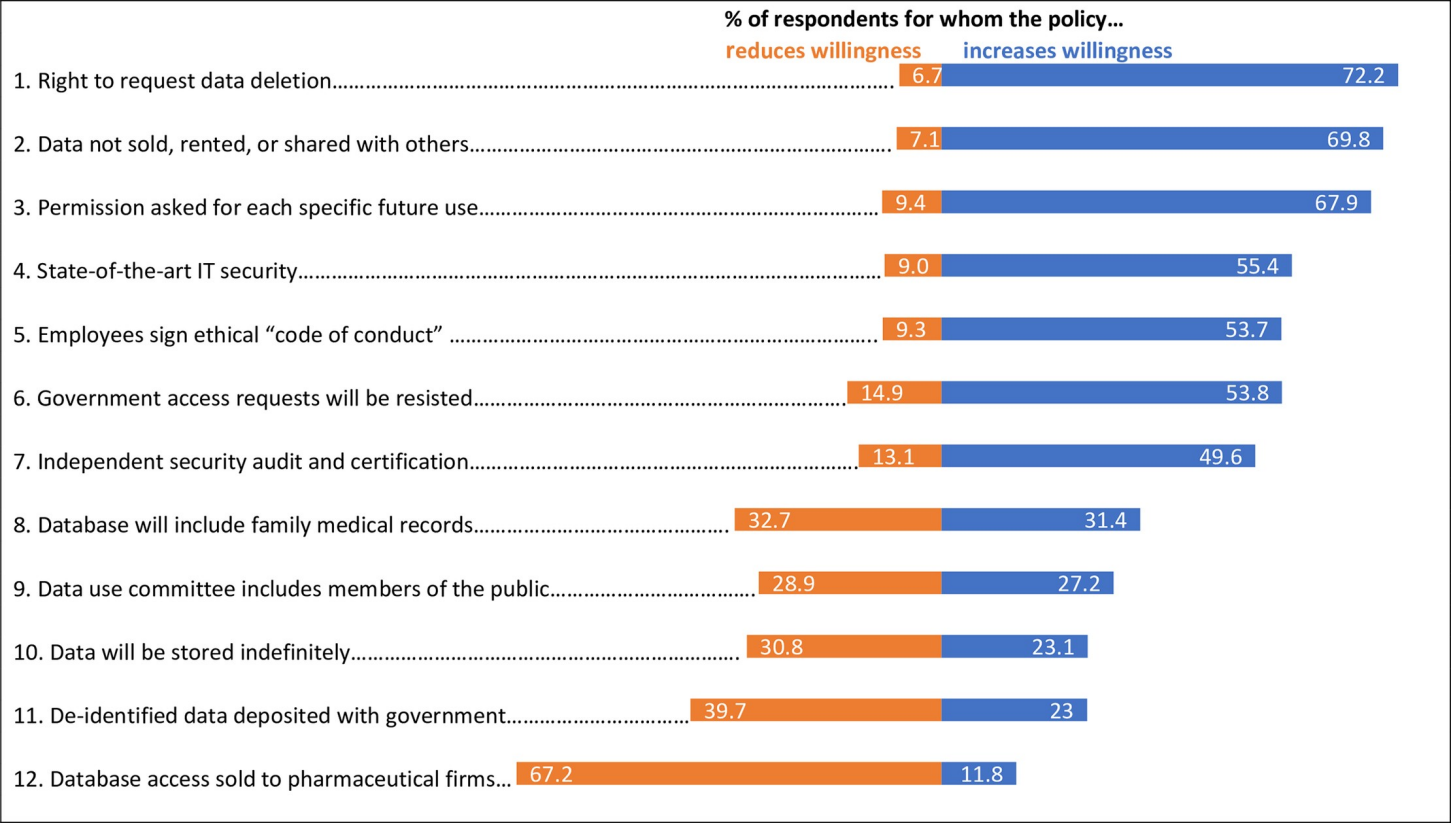
Effects of genomic data governance policies on respondent’s willingness to provide data. Values for each policy do not add up to 100% because they omit those whose feelings toward it were neutral.

Our survey also asked respondents how twelve specific governance policies affect their willingness to provide genomic data. Results are displayed in Fig 2. In this figure, red bars indicate the portion of respondents for whom each governance policy would decrease their willingness to provide data, while green bars indicate the portion of respondents for whom that policy would increase their willingness. Governance policies are listed in order from those that increase willingness the most on top, to those that decrease willingness the most on bottom. The three policies that increased willingness the most were: 1) the ability for data providers to request that their data be deleted; 2) assurance that providers’ data will not be sold or shared beyond the organization collecting it; and 3) re-use of providers’ data will require specific permissions. The three policies that decreased willingness the most were: 1) selling database access to pharmaceutical firms; 2) providing data to the federal government; and 3) retaining the data indefinitely without a specified date for destruction.

In their attitudes about these genomic governance policies, ‘donor’ respondents tended to resemble ‘seller’ respondents, except that their willingness was even more strongly reduced by three of the most willingness-decreasing policies (policies 8, 10 and 11 in **S1 Appendix**). Perhaps not surprisingly, ‘unwilling’ respondents were categorically more negative than both ‘donor’ and ‘seller’ respondents in their reactions to all the policies.

A common denominator across these governance policy findings is a preference for restrictions on sharing or reuse, unless permission is specifically granted by the individual. These preferences appear to pose a challenge for the goals and business models of many database-owning organizations, which often envision that their databases will serve multiple, not-necessarily-specified scientific and commercial purposes, through access arrangements with multiple outside partners. This tension appears to hold equally for commercial as well as public organizations [17].

### Effects of database-owning organization type and sector

In the GDGS survey, public attitudes did not appear to depend much on the type of organization owning the genomic database. All respondents were randomly assigned to consider one of five different types of organizations requesting their genomic data (technology company, hospital organization, university lab, pharmaceutical firm, government agency). The rate of ‘donor’ respondents was only slightly higher for hospital (14.5%), university (14.0%), and government (12.5%), compared with technology (10.9%) and pharmaceutical (6.19%) settings. In addition, only one of the twelve governance policies (policy 7 in S2 Appendix) yielded a significant difference across the organization types. These findings indicate that public perceptions of the risks and benefits of providing genomic data do not depend much on the sector making the data request. Hospitals and universities, commonly associated with stronger social missions compared with commercial businesses, only had slightly higher ‘donor’ rates, and did not differ in terms of respondents’ governance policy priorities.

Among ‘seller’ respondents, compensation expectation levels (with or without bundled report) did not differ across organization types. Using unadjusted values, Wilks’ lambda statistic = 0.9948 (F(1.34), Pr = 0.2526) for payment, and 0.9947 (F(1.34), Pr = 0.2522) for a bundled transaction. Using log(10)-transformed values as shown in Fig 1, Wilks’ lambda statistic = 0.9956 (F(1.11), Pr = 0.3498) for payment, and 0.9966 (F(0.88), Pr = 0.4769) for a bundled transaction.

## Discussion

Overall, these findings indicate that as public awareness grows regarding the commercial aspects and privacy issues of genomic databases, Individuals’ expectations for compensation in exchange for data provision may rise. Once confronted with publicly available, factual information from mainstream news reports, most respondents reported preferring governance policies that would give them more control over their data. These expectations and concerns are also relatively consistent across organizational contexts and sectors.

Based on these findings, it seems unlikely that a one-size-fits-all approach will meet public expectations for responsible genomic database governance. Most important, a majority of respondents would clearly prefer a more transparent and participant-centric approach, giving more control, confidence, and compensation to those individuals providing their data. Although such participant-centric policies complicate database use, recent technological and organizational advances increase their feasibility. For example, technological solutions are being pursued that might give individual data providers more control of who accesses and uses their data and what their data are used for–while at the same time still allowing scalable data sharing to meet the needs of data users across health care, academic, and industry communities.

Ongoing independent research is needed to track evolving public attitudes regarding genomic databases. Currently, surveys on this topic are typically sponsored by institutions that own or support genomic biobanks. These institutions are often familiar to the respondents queried. Thus those studies have been beneficial in informing those specific institutions how one group stakeholders (patients and local community) think about biobanking and the institution’s use of their genomic sequence data. Our approach, however, was to explore willingness in the context of a new era in which personal information, including genetic data is the ‘new oil’–i.e. a substance of tremendous societal and commercial value. However, our results offer a forward-looking window on individual preferences that can be useful for institutions of all types as they develop governance approaches for the modern era of large-scale data sharing.

### Limitations

While we intentionally primed participants with information on the commercial value of genomic sequence data, in order to begin to answer our research question, this priming does limit the open-mindedness with which participants approached the survey questions. In addition, the organizational type descriptions we used have limitations. We did not specify if GreatCare Hospital is meant to be familiar and local to the participant. As a result, we do not know how participants were considering the organization, relative to prior studies that made such an association. Similarly, for the University organization type, Middle State University was not identified as a known entity to the participant. Local proximity could be important for engendering trust and willingness to donate.

## Supporting information

S1 Appendix Transcript of informational priming slides. (DOCX)

S2 Appendix Genomic data governance policy statements. (DOCX)

S3 Appendix Frequency tables. (DOCX)
