# Supplementary material for: Evolving public views on the value of one’s DNA and expectations for genomic database governance: Results from a national survey
Source: PLoS One. 2020 Mar 11;15(3):e0229044. doi: 10.1371/journal.pone.0229044 (PMC7065739; doi:10.1371/journal.pone.0229044)
Supplement: S1 Appendix — (DOCX) [file pone.0229044.s001.docx]

**S1 Appendix**

**Transcript of informational priming slides**

**Recent and Future Trends with DNA Data**

- Today your unique DNA sequence can be rapidly generated and digitally stored, just from saliva, for about $100
- When data from many people are combined together, the resulting DNA database can be used for many different purposes. For example:
  - medical research (e.g. finding new pharmaceutical drugs)
  - health care (e.g. testing for risk of breast cancer)
  - family ancestry research
  - citizen identity verification
  - crime-scene investigation
  - and more

**DNA data also reveal a lot about you**

- These databases can be used to predict physical and mental risks for each individual. For example:
  - Risks of depression, dementia, and having children with birth defects
- Your risks can also be discovered from your relative’s data
  - Even if your own data is missing
- The databases can also be used to prove (or disprove) paternity
- And to catch criminals. For example:
  - The cold-case “Golden State Killer” was recently caught using a commercial database that happened to include DNA from his relative

**What is the financial value of DNA data?**

- As these databases prove useful, their financial value is being realized
  - Bigger is better: Some have millions of people’s DNA in them
  - Many combine DNA with medical records & personal information
- Companies and hospitals are starting to sell and rent access to DNA databases
- These transactions suggest companies may be willing to pay $130 per sample for database access (Reuters 2018)

**Providing your DNA data**

- Currently, some organizations will pay you to obtain your DNA data
- Others offer to obtain it in exchange for a personalized report on your health risks and ancestry information
- Still others ask you to donate it for free, as a charitable act that contributes to medical research
- Regardless of how they obtain it, most organizations agree to attempt to protect the data and your privacy
- You also must sign a legal document acknowledging that you understand there are still privacy risks; your data could still be stolen or misused
