# Supplementary material for: Evolving public views on the value of one’s DNA and expectations for genomic database governance: Results from a national survey
Source: PLoS One. 2020 Mar 11;15(3):e0229044. doi: 10.1371/journal.pone.0229044 (PMC7065739; doi:10.1371/journal.pone.0229044)
Supplement: S2 Appendix — (DOCX) [file pone.0229044.s002.docx]

**S2 Appendix**

**Genomic data governance policy statements**

Respondents were asked how each of the following twelve policies would affect their willingness to provide their genomic data. Questions were asked in a randomized order to reduce potential question order bias.

1. Individuals have the right to request that their DNA data be deleted from the database at any time
2. DNA data are not sold, rented, or shared with any other organizations
3. Individuals will be asked permission for each specific use of their DNA data in the future
4. State-of-the-art IT security are used for all DNA data and other customer data
5. All employees sign an ethical “code of conduct” which includes safeguarding of DNA data
6. Government requests for access to DNA data are refused without a warrant
7. An independent company audits and certifies DNA data security
8. Access to family medical records is required, and these will be linked to DNA data
9. Members of the general public serve on a committee to decide how genomic data will be used
10. Individuals’ DNA data are stored indefinitely
11. Copies of all DNA data (without individuals’ names) are deposited into a government database
12. Access to DNA data is sold to pharmaceutical firms (without requesting further permission)
