# Supplementary material for: Evolving public views on the value of one’s DNA and expectations for genomic database governance: Results from a national survey
Source: PLoS One. 2020 Mar 11;15(3):e0229044. doi: 10.1371/journal.pone.0229044 (PMC7065739; doi:10.1371/journal.pone.0229044)
Supplement: S3 Appendix — (DOCX) [file pone.0229044.s003.docx]

| **S3 Appendix**  **Frequency Tables**  **Frequency Tables 1.1-1.12** |
| --- |
| Effects of Governance Policies on Willingness to Provide Data: |
| Comparisons across Donor, Seller, and Unwilling Respondents |
|  |
| Results of two-group Wilcoxon rank-sum (Mann-Whitney) test for each governance policy |
| are provided below each corresponding table. |

| **Table 1.1** |  |  |  |  |  |  |
| --- | --- | --- | --- | --- | --- | --- |
| Policy: | Individuals have the right to request that their DNA data be deleted from the database at any time | | | | | |
|  |  |  |  |  |  |  |
|  | Reduce greatly | Reduce a little | No effect | Increase a little | Increase greatly | Total |
| Unwilling | 56 | 30 | 227 | 270 | 177 | 760 |
|  | 7.37 | 3.95 | 29.87 | 35.53 | 23.29 | 100.00 |
|  | 71.79 | 52.63 | 53.29 | 44.26 | 20.95 | 37.70 |
| Sellers | 19 | 21 | 155 | 282 | 545 | 1022 |
|  | 1.86 | 2.05 | 15.17 | 27.59 | 53.33 | 100.00 |
|  | 24.36 | 36.84 | 36.38 | 46.23 | 64.50 | 50.69 |
| Donors | 3 | 6 | 44 | 58 | 123 | 234 |
|  | 1.28 | 2.56 | 18.80 | 24.79 | 52.56 | 100.00 |
|  | 3.85 | 10.53 | 10.33 | 9.51 | 14.56 | 11.61 |
| Total | 78 | 57 | 426 | 610 | 845 | 2016 |
|  | 3.87 | 2.83 | 21.13 | 30.26 | 41.91 | 100.00 |
|  | 100.00 | 100.00 | 100.00 | 100.00 | 100.00 | 100.00 |
|  |  |  |  |  |  |  |
|  | Two-sample Wilcoxon rank-sum (Mann-Whitney) tests | | | | |  |
|  | z = 0.555 | | Ho: (seller) = (donor) | |  |  |
|  | Prob > \|z\| = 0.5792 | |  |  |  |  |
|  |  |  |  |  |  |  |
|  | z = -7.876 | | Ho: (unwilling) = (donor) | | |  |
|  | Prob > \|z\| = 0.0000 | |  |  |  |  |
|  |  |  |  |  |  |  |
|  | z = -13.478 | | Ho: (unwilling) = (seller) | | |  |
|  | Prob > \|z\| = 0.0000 | |  |  |  |  |
|  |  |  |  |  |  |  |

| **Table 1.2** |  |  |  |  |  |  |
| --- | --- | --- | --- | --- | --- | --- |
| Policy: | DNA data are not sold, rented, or shared with any other organizations | | | | | |
|  |  |  |  |  |  |  |
|  | Reduce greatly | Reduce a little | No effect | Increase a little | Increase greatly | Total |
| Unwilling | 70 | 29 | 262 | 287 | 112 | 760 |
|  | 9.21 | 3.82 | 34.47 | 37.76 | 14.74 | 100.00 |
|  | 78.65 | 53.70 | 56.22 | 39.53 | 16.45 | 37.70 |
| Sellers | 17 | 20 | 155 | 381 | 449 | 1022 |
|  | 1.66 | 1.96 | 15.17 | 37.28 | 43.93 | 100.00 |
|  | 19.10 | 37.04 | 33.26 | 52.48 | 65.93 | 50.69 |
| Donors | 2 | 5 | 49 | 58 | 120 | 234 |
|  | 0.85 | 2.14 | 20.94 | 24.79 | 51.28 | 100.00 |
|  | 2.25 | 9.26 | 10.52 | 7.99 | 17.62 | 11.61 |
| Total | 89 | 54 | 466 | 726 | 681 | 2016 |
|  | 4.41 | 2.68 | 23.12 | 36.01 | 33.78 | 100.00 |
|  | 100.00 | 100.00 | 100.00 | 100.00 | 100.00 | 100.00 |
|  |  |  |  |  |  |  |
|  | Two-sample Wilcoxon rank-sum (Mann-Whitney) tests | | | | |  |
|  | z = -0.878 | | Ho: (seller) = (donor) | |  |  |
|  | Prob > \|z\| = 0.3802 | |  |  |  |  |
|  |  |  |  |  |  |  |
|  | z = -9.963 | | Ho: (unwilling) = (donor) | | |  |
|  | Prob > \|z\| = 0.0000 | |  |  |  |  |
|  |  |  |  |  |  |  |
|  | z = -15.465 | | Ho: (unwilling) = (seller) | | |  |
|  | Prob > \|z\| = 0.0000 | |  |  |  |  |
|  |  |  |  |  |  |  |

| **Table 1.3** |  |  |  |  |  |  |
| --- | --- | --- | --- | --- | --- | --- |
| Policy: | Individuals will be asked permission for each specific use of their DNA data in the future | | | | | |
|  |  |  |  |  |  |  |
|  | Reduce greatly | Reduce a little | No effect | Increase a little | Increase greatly | Total |
| Unwilling | 73 | 53 | 252 | 278 | 104 | 760 |
|  | 9.61 | 6.97 | 33.16 | 36.58 | 13.68 | 100.00 |
|  | 82.95 | 51.96 | 55.14 | 38.99 | 15.85 | 37.70 |
| Sellers | 13 | 39 | 162 | 371 | 437 | 1022 |
|  | 1.27 | 3.82 | 15.85 | 36.30 | 42.76 | 100.00 |
|  | 14.77 | 38.24 | 35.45 | 52.03 | 66.62 | 50.69 |
| Donors | 2 | 10 | 43 | 64 | 115 | 234 |
|  | 0.85 | 4.27 | 18.38 | 27.35 | 49.15 | 100.00 |
|  | 2.27 | 9.80 | 9.41 | 8.98 | 17.53 | 11.61 |
| Total | 88 | 102 | 457 | 713 | 656 | 2016 |
|  | 4.37 | 5.06 | 22.67 | 35.37 | 32.54 | 100.00 |
|  | 100.00 | 100.00 | 100.00 | 100.00 | 100.00 | 100.00 |
|  |  |  |  |  |  |  |
|  | Two-sample Wilcoxon rank-sum (Mann-Whitney) tests | | | | |  |
|  | z = -0.954 | | Ho: (seller) = (donor) | |  |  |
|  | Prob > \|z\| = 0.3399 | |  |  |  |  |
|  |  |  |  |  |  |  |
|  | z = -10.169 | | Ho: (unwilling) = (donor) | | |  |
|  | Prob > \|z\| = 0.0000 | |  |  |  |  |
|  |  |  |  |  |  |  |
|  | z = -15.412 | | Ho: (unwilling) = (seller) | | |  |
|  | Prob > \|z\| = 0.0000 | |  |  |  |  |
|  |  |  |  |  |  |  |

| Table 1.4 |  |  |  |  |  |  |
| --- | --- | --- | --- | --- | --- | --- |
| Policy: | State-of-the-art IT security are used for all DNA data and other customer data | | | | | |
|  |  |  |  |  |  |  |
|  | Reduce greatly | Reduce a little | No effect | Increase a little | Increase greatly | Total |
| Unwilling | 70 | 45 | 383 | 206 | 56 | 760 |
|  | 9.21 | 5.92 | 50.39 | 27.11 | 7.37 | 100.00 |
|  | 77.78 | 48.91 | 53.42 | 31.84 | 11.91 | 37.70 |
| Sellers | 16 | 38 | 271 | 385 | 312 | 1022 |
|  | 1.57 | 3.72 | 26.52 | 37.67 | 30.53 | 100.00 |
|  | 17.78 | 41.30 | 37.80 | 59.51 | 66.38 | 50.69 |
| Donors | 4 | 9 | 63 | 56 | 102 | 234 |
|  | 1.71 | 3.85 | 26.92 | 23.93 | 43.59 | 100.00 |
|  | 4.44 | 9.78 | 8.79 | 8.66 | 21.70 | 11.61 |
| Total | 90 | 92 | 717 | 647 | 470 | 2016 |
|  | 4.46 | 4.56 | 35.57 | 32.09 | 23.31 | 100.00 |
|  | 100.00 | 100.00 | 100.00 | 100.00 | 100.00 | 100.00 |
|  |  |  |  |  |  |  |
|  | Two-sample Wilcoxon rank-sum (Mann-Whitney) tests | | | | |  |
|  | z = -2.109 | | Ho: (seller) = (donor) | |  |  |
|  | Prob > \|z\| = 0.0350 | |  |  |  |  |
|  |  |  |  |  |  |  |
|  | z = -10.974 | | Ho: (unwilling) = (donor) | | |  |
|  | Prob > \|z\| = 0.0000 | |  |  |  |  |
|  |  |  |  |  |  |  |
|  | z = -15.513 | | Ho: (unwilling) = (seller) | | |  |
|  | Prob > \|z\| = 0.0000 | |  |  |  |  |
|  |  |  |  |  |  |  |

| **Table 1.5** |  |  |  |  |  |  |
| --- | --- | --- | --- | --- | --- | --- |
| Policy: | All employees sign an ethical “code of conduct” which includes safeguarding of DNA data | | | | | |
|  |  |  |  |  |  |  |
|  | Reduce greatly | Reduce a little | No effect | Increase a little | Increase greatly | Total |
| Unwilling | 82 | 39 | 432 | 153 | 54 | 760 |
|  | 10.79 | 5.13 | 56.84 | 20.13 | 7.11 | 100.00 |
|  | 77.36 | 47.56 | 57.99 | 25.50 | 11.18 | 37.70 |
| Sellers | 21 | 35 | 254 | 387 | 325 | 1022 |
|  | 2.05 | 3.42 | 24.85 | 37.87 | 31.80 | 100.00 |
|  | 19.81 | 42.68 | 34.09 | 64.50 | 67.29 | 50.69 |
| Donors | 3 | 8 | 59 | 60 | 104 | 234 |
|  | 1.28 | 3.42 | 25.21 | 25.64 | 44.44 | 100.00 |
|  | 2.83 | 9.76 | 7.92 | 10.00 | 21.53 | 11.61 |
| Total | 106 | 82 | 745 | 600 | 483 | 2016 |
|  | 5.26 | 4.07 | 36.95 | 29.76 | 23.96 | 100.00 |
|  | 100.00 | 100.00 | 100.00 | 100.00 | 100.00 | 100.00 |
|  |  |  |  |  |  |  |
|  | Two-sample Wilcoxon rank-sum (Mann-Whitney) tests | | | | |  |
|  | z = -2.351 | | Ho: (seller) = (donor) | |  |  |
|  | Prob > \|z\| = 0.0187 | |  |  |  |  |
|  |  |  |  |  |  |  |
|  | z = -12.853 | | Ho: (unwilling) = (donor) | | |  |
|  | Prob > \|z\| = 0.0000 | |  |  |  |  |
|  |  |  |  |  |  |  |
|  | z = -17.952 | | Ho: (unwilling) = (seller) | | |  |
|  | Prob > \|z\| = 0.0000 | |  |  |  |  |
|  |  |  |  |  |  |  |

| **Table 1.6** |  |  |  |  |  |  |
| --- | --- | --- | --- | --- | --- | --- |
| Policy: | Government requests for access to DNA data are refused without a warrant | | | | | |
|  |  |  |  |  |  |  |
|  | Reduce greatly | Reduce a little | No effect | Increase a little | Increase greatly | Total |
| Unwilling | 109 | 49 | 322 | 209 | 71 | 760 |
|  | 14.34 | 6.45 | 42.37 | 27.50 | 9.34 | 100.00 |
|  | 60.56 | 40.83 | 51.03 | 33.60 | 15.33 | 37.70 |
| Sellers | 57 | 58 | 243 | 352 | 312 | 1022 |
|  | 5.58 | 5.68 | 23.78 | 34.44 | 30.53 | 100.00 |
|  | 31.67 | 48.33 | 38.51 | 56.59 | 67.39 | 50.69 |
| Donors | 14 | 13 | 66 | 61 | 80 | 234 |
|  | 5.98 | 5.56 | 28.21 | 26.07 | 34.19 | 100.00 |
|  | 7.78 | 10.83 | 10.46 | 9.81 | 17.28 | 11.61 |
| Total | 180 | 120 | 631 | 622 | 463 | 2016 |
|  | 8.93 | 5.95 | 31.30 | 30.85 | 22.97 | 100.00 |
|  | 100.00 | 100.00 | 100.00 | 100.00 | 100.00 | 100.00 |
|  |  |  |  |  |  |  |
|  | Two-sample Wilcoxon rank-sum (Mann-Whitney) tests | | | | |  |
|  | z = 0.124 | | Ho: (seller) = (donor) | |  |  |
|  | Prob > \|z\| = 0.9015 | |  |  |  |  |
|  |  |  |  |  |  |  |
|  | z = -7.691 | | Ho: (unwilling) = (donor) | | |  |
|  | Prob > \|z\| = 0.0000 | |  |  |  |  |
|  |  |  |  |  |  |  |
|  | z = -12.749 | | Ho: (unwilling) = (seller) | | |  |
|  | Prob > \|z\| = 0.0000 | |  |  |  |  |
|  |  |  |  |  |  |  |

| **Table 1.7** |  |  |  |  |  |  |
| --- | --- | --- | --- | --- | --- | --- |
| Policy: | An independent company audits and certifies DNA data security | | | | | |
|  |  |  |  |  |  |  |
|  | Reduce greatly | Reduce a little | No effect | Increase a little | Increase greatly | Total |
| Unwilling | 93 | 53 | 370 | 190 | 54 | 760 |
|  | 12.24 | 6.97 | 48.68 | 25.00 | 7.11 | 100.00 |
|  | 73.23 | 38.41 | 49.27 | 31.56 | 13.57 | 37.70 |
| Sellers | 28 | 71 | 311 | 344 | 268 | 1022 |
|  | 2.74 | 6.95 | 30.43 | 33.66 | 26.22 | 100.00 |
|  | 22.05 | 51.45 | 41.41 | 57.14 | 67.34 | 50.69 |
| Donors | 6 | 14 | 70 | 68 | 76 | 234 |
|  | 2.56 | 5.98 | 29.91 | 29.06 | 32.48 | 100.00 |
|  | 4.72 | 10.14 | 9.32 | 11.30 | 19.10 | 11.61 |
| Total | 127 | 138 | 751 | 602 | 398 | 2016 |
|  | 6.30 | 6.85 | 37.25 | 29.86 | 19.74 | 100.00 |
|  | 100.00 | 100.00 | 100.00 | 100.00 | 100.00 | 100.00 |
|  |  |  |  |  |  |  |
|  | Two-sample Wilcoxon rank-sum (Mann-Whitney) tests | | | | |  |
|  | z = -1.311 | | Ho: (seller) = (donor) | |  |  |
|  | Prob > \|z\| = 0.1900 | |  |  |  |  |
|  |  |  |  |  |  |  |
|  | z = -9.224 | | Ho: (unwilling) = (donor) | | |  |
|  | Prob > \|z\| = 0.0000 | |  |  |  |  |
|  |  |  |  |  |  |  |
|  | z = -12.777 | | Ho: (unwilling) = (seller) | | |  |
|  | Prob > \|z\| = 0.0000 | |  |  |  |  |
|  |  |  |  |  |  |  |

| **Table 1.8** |  |  |  |  |  |  |
| --- | --- | --- | --- | --- | --- | --- |
| Policy: | Access to family medical records is required, and these will be linked to DNA data | | | | | |
|  |  |  |  |  |  |  |
|  | Reduce greatly | Reduce a little | No effect | Increase a little | Increase greatly | Total |
| Unwilling | 199 | 142 | 288 | 104 | 27 | 760 |
|  | 26.18 | 18.68 | 37.89 | 13.68 | 3.55 | 100.00 |
|  | 64.82 | 40.23 | 39.89 | 26.80 | 10.98 | 37.70 |
| Sellers | 90 | 182 | 358 | 234 | 158 | 1022 |
|  | 8.81 | 17.81 | 35.03 | 22.90 | 15.46 | 100.00 |
|  | 29.32 | 51.56 | 49.58 | 60.31 | 64.23 | 50.69 |
| Donors | 18 | 29 | 76 | 50 | 61 | 234 |
|  | 7.69 | 12.39 | 32.48 | 21.37 | 26.07 | 100.00 |
|  | 5.86 | 8.22 | 10.53 | 12.89 | 24.80 | 11.61 |
| Total | 307 | 353 | 722 | 388 | 246 | 2016 |
|  | 15.23 | 17.51 | 35.81 | 19.25 | 12.20 | 100.00 |
|  | 100.00 | 100.00 | 100.00 | 100.00 | 100.00 | 100.00 |
|  |  |  |  |  |  |  |
|  | Two-sample Wilcoxon rank-sum (Mann-Whitney) tests | | | | |  |
|  | z = -3.228 | | Ho: (seller) = (donor) | |  |  |
|  | Prob > \|z\| = 0.0012 | |  |  |  |  |
|  |  |  |  |  |  |  |
|  | z = -10.026 | | Ho: (unwilling) = (donor) | | |  |
|  | Prob > \|z\| = 0.0000 | |  |  |  |  |
|  |  |  |  |  |  |  |
|  | z = -11.621 | | Ho: (unwilling) = (seller) | | |  |
|  | Prob > \|z\| = 0.0000 | |  |  |  |  |
|  |  |  |  |  |  |  |

| **Table 1.9** |  |  |  |  |  |  |
| --- | --- | --- | --- | --- | --- | --- |
| Policy: | Members of the general public serve on a committee to decide how genomic data will be used | | | | | |
|  |  |  |  |  |  |  |
|  | Reduce greatly | Reduce a little | No effect | Increase a little | Increase greatly | Total |
| Unwilling | 169 | 111 | 366 | 98 | 16 | 760 |
|  | 22.24 | 14.61 | 48.16 | 12.89 | 2.11 | 100.00 |
|  | 60.36 | 36.75 | 41.31 | 26.20 | 9.20 | 37.70 |
| Sellers | 91 | 155 | 435 | 226 | 115 | 1022 |
|  | 8.90 | 15.17 | 42.56 | 22.11 | 11.25 | 100.00 |
|  | 32.50 | 51.32 | 49.10 | 60.43 | 66.09 | 50.69 |
| Donors | 20 | 36 | 85 | 50 | 43 | 234 |
|  | 8.55 | 15.38 | 36.32 | 21.37 | 18.38 | 100.00 |
|  | 7.14 | 11.92 | 9.59 | 13.37 | 24.71 | 11.61 |
| Total | 280 | 302 | 886 | 374 | 174 | 2016 |
|  | 13.89 | 14.98 | 43.95 | 18.55 | 8.63 | 100.00 |
|  | 100.00 | 100.00 | 100.00 | 100.00 | 100.00 | 100.00 |
|  |  |  |  |  |  |  |
|  | Two-sample Wilcoxon rank-sum (Mann-Whitney) tests | | | | |  |
|  | z = -1.668 | | Ho: (seller) = (donor) | |  |  |
|  | Prob > \|z\| = 0.0953 | |  |  |  |  |
|  |  |  |  |  |  |  |
|  | z = -7.521 | | Ho: (unwilling) = (donor) | | |  |
|  | Prob > \|z\| = 0.0000 | |  |  |  |  |
|  |  |  |  |  |  |  |
|  | z = -9.742 | | Ho: (unwilling) = (seller) | | |  |
|  | Prob > \|z\| = 0.0000 | |  |  |  |  |
|  |  |  |  |  |  |  |

| **Table 1.10** |  |  |  |  |  |  |
| --- | --- | --- | --- | --- | --- | --- |
| Policy: | Individuals’ DNA data are stored indefinitely | | | |  |  |
|  |  |  |  |  |  |  |
|  | Reduce greatly | Reduce a little | No effect | Increase a little | Increase greatly | Total |
| Unwilling | 227 | 131 | 330 | 53 | 19 | 760 |
|  | 29.87 | 17.24 | 43.42 | 6.97 | 2.50 | 100.00 |
|  | 78.01 | 39.82 | 35.45 | 19.49 | 9.84 | 37.70 |
| Sellers | 62 | 171 | 481 | 182 | 126 | 1022 |
|  | 6.07 | 16.73 | 47.06 | 17.81 | 12.33 | 100.00 |
|  | 21.31 | 51.98 | 51.66 | 66.91 | 65.28 | 50.69 |
| Donors | 2 | 27 | 120 | 37 | 48 | 234 |
|  | 0.85 | 11.54 | 51.28 | 15.81 | 20.51 | 100.00 |
|  | 0.69 | 8.21 | 12.89 | 13.60 | 24.87 | 11.61 |
| Total | 291 | 329 | 931 | 272 | 193 | 2016 |
|  | 14.43 | 16.32 | 46.18 | 13.49 | 9.57 | 100.00 |
|  | 100.00 | 100.00 | 100.00 | 100.00 | 100.00 | 100.00 |
|  |  |  |  |  |  |  |
|  | Two-sample Wilcoxon rank-sum (Mann-Whitney) tests | | | | |  |
|  | z = -3.647 | | Ho: (seller) = (donor) | |  |  |
|  | Prob > \|z\| = 0.0003 | |  |  |  |  |
|  |  |  |  |  |  |  |
|  | z = -12.264 | | Ho: (unwilling) = (donor) | | |  |
|  | Prob > \|z\| = 0.0000 | |  |  |  |  |
|  |  |  |  |  |  |  |
|  | z = -14.263 | | Ho: (unwilling) = (seller) | | |  |
|  | Prob > \|z\| = 0.0000 | |  |  |  |  |
|  |  |  |  |  |  |  |

| **Table 1.11** |  |  |  |  |  |  |
| --- | --- | --- | --- | --- | --- | --- |
| Policy: | Copies of all DNA data (without individuals’ names) are deposited into a government database | | | | | |
|  |  |  |  |  |  |  |
|  | Reduce greatly | Reduce a little | No effect | Increase a little | Increase greatly | Total |
| Unwilling | 272 | 130 | 257 | 69 | 32 | 760 |
|  | 35.79 | 17.11 | 33.82 | 9.08 | 4.21 | 100.00 |
|  | 64.61 | 34.30 | 34.13 | 25.27 | 16.84 | 37.70 |
| Sellers | 131 | 213 | 404 | 163 | 111 | 1022 |
|  | 12.82 | 20.84 | 39.53 | 15.95 | 10.86 | 100.00 |
|  | 31.12 | 56.20 | 53.65 | 59.71 | 58.42 | 50.69 |
| Donors | 18 | 36 | 92 | 41 | 47 | 234 |
|  | 7.69 | 15.38 | 39.32 | 17.52 | 20.09 | 100.00 |
|  | 4.28 | 9.50 | 12.22 | 15.02 | 24.74 | 11.61 |
| Total | 421 | 379 | 753 | 273 | 190 | 2016 |
|  | 20.88 | 18.80 | 37.35 | 13.54 | 9.42 | 100.00 |
|  | 100.00 | 100.00 | 100.00 | 100.00 | 100.00 | 100.00 |
|  |  |  |  |  |  |  |
|  | Two-sample Wilcoxon rank-sum (Mann-Whitney) tests | | | | |  |
|  | z = -4.123 | | Ho: (seller) = (donor) | |  |  |
|  | Prob > \|z\| = 0.0000 | |  |  |  |  |
|  |  |  |  |  |  |  |
|  | z = -10.251 | | Ho: (unwilling) = (donor) | | |  |
|  | Prob > \|z\| = 0.0000 | |  |  |  |  |
|  |  |  |  |  |  |  |
|  | z = -10.746 | | Ho: (unwilling) = (seller) | | |  |
|  | Prob > \|z\| = 0.0000 | |  |  |  |  |
|  |  |  |  |  |  |  |

| **Table 1.12** |  |  |  |  |  |  |
| --- | --- | --- | --- | --- | --- | --- |
| Policy: | Access to DNA data is sold to pharmaceutical firms (without requesting further permission) | | | | | |
|  |  |  |  |  |  |  |
|  | Reduce greatly | Reduce a little | No effect | Increase a little | Increase greatly | Total |
| Unwilling | 457 | 104 | 147 | 35 | 17 | 760 |
|  | 60.13 | 13.68 | 19.34 | 4.61 | 2.24 | 100.00 |
|  | 52.05 | 21.85 | 34.59 | 26.72 | 16.04 | 37.70 |
| Sellers | 350 | 307 | 219 | 82 | 64 | 1022 |
|  | 34.25 | 30.04 | 21.43 | 8.02 | 6.26 | 100.00 |
|  | 39.86 | 64.50 | 51.53 | 62.60 | 60.38 | 50.69 |
| Donors | 71 | 65 | 59 | 14 | 25 | 234 |
|  | 30.34 | 27.78 | 25.21 | 5.98 | 10.68 | 100.00 |
|  | 8.09 | 13.66 | 13.88 | 10.69 | 23.58 | 11.61 |
| Total | 878 | 476 | 425 | 131 | 106 | 2016 |
|  | 43.55 | 23.61 | 21.08 | 6.50 | 5.26 | 100.00 |
|  | 100.00 | 100.00 | 100.00 | 100.00 | 100.00 | 100.00 |
|  |  |  |  |  |  |  |
|  | Two-sample Wilcoxon rank-sum (Mann-Whitney) tests | | | | |  |
|  | z = -1.743 | | Ho: (seller) = (donor) | |  |  |
|  | Prob > \|z\| = 0.0813 | |  |  |  |  |
|  |  |  |  |  |  |  |
|  | z = -7.624 | | Ho: (unwilling) = (donor) | | |  |
|  | Prob > \|z\| = 0.0000 | |  |  |  |  |
|  |  |  |  |  |  |  |
|  | z = -9.339 | | Ho: (unwilling) = (seller) | | |  |
|  | Prob > \|z\| = 0.0000 | |  |  |  |  |

| **Frequency Tables 2.1-2.12** |
| --- |
| Effects of Governance Policies on Willingness to Provide Data: |
| Comparisons across Five Randomly-Assigned Types of Organizations |
|  |
| Results of multi-group Kruskal-Wallis equality-of-populations rank test for each governance policy |
| are provided below each corresponding table. |

| **Table 2.1** |  |  |  |  |  |  |
| --- | --- | --- | --- | --- | --- | --- |
| Policy: | Individuals have the right to request that their DNA data be deleted from the database at any time | | | | | |
|  |  |  |  |  |  |  |
|  | Reduce greatly | Reduce a little | No effect | Increase a little | Increase greatly | Total |
| Tech. | 11 | 8 | 100 | 126 | 159 | 404 |
|  | 2.72 | 1.98 | 24.75 | 31.19 | 39.36 | 100.00 |
|  | 14.10 | 14.04 | 23.47 | 20.66 | 18.82 | 20.04 |
| Hosp. | 19 | 8 | 84 | 114 | 167 | 392 |
|  | 4.85 | 2.04 | 21.43 | 29.08 | 42.60 | 100.00 |
|  | 24.36 | 14.04 | 19.72 | 18.69 | 19.76 | 19.44 |
| Univ. | 15 | 14 | 79 | 114 | 178 | 400 |
|  | 3.75 | 3.50 | 19.75 | 28.50 | 44.50 | 100.00 |
|  | 19.23 | 24.56 | 18.54 | 18.69 | 21.07 | 19.84 |
| Pharm. | 13 | 13 | 74 | 135 | 168 | 403 |
|  | 3.23 | 3.23 | 18.36 | 33.50 | 41.69 | 100.00 |
|  | 16.67 | 22.81 | 17.37 | 22.13 | 19.88 | 19.99 |
| Govt. | 20 | 14 | 89 | 121 | 173 | 417 |
|  | 4.80 | 3.36 | 21.34 | 29.02 | 41.49 | 100.00 |
|  | 25.64 | 24.56 | 20.89 | 19.84 | 20.47 | 20.68 |
| Total | 78 | 57 | 426 | 610 | 845 | 2016 |
|  | 3.87 | 2.83 | 21.13 | 30.26 | 41.91 | 100.00 |
|  | 100.00 | 100.00 | 100.00 | 100.00 | 100.00 | 100.00 |
|  |  |  |  |  |  |  |
|  | Kruskal-Wallis equality-of-populations rank test | | | | |  |
|  | chi-squared = 1.692 with 4 d.f. | | |  |  |  |
|  | probability = 0.7922 | |  |  |  |  |
|  |  |  |  |  |  |  |
|  | chi-squared with ties = 1.903 with 4 d.f. | | | |  |  |
|  | probability = 0.7536 | |  |  |  |  |
|  |  |  |  |  |  |  |

| **Table 2.2** |  |  |  |  |  |  |
| --- | --- | --- | --- | --- | --- | --- |
| Policy: | DNA data are not sold, rented, or shared with any other organizations | | | | | |
|  |  |  |  |  |  |  |
|  | Reduce greatly | Reduce a little | No effect | Increase a little | Increase greatly | Total |
| Tech. | 12 | 11 | 102 | 155 | 124 | 404 |
|  | 2.97 | 2.72 | 25.25 | 38.37 | 30.69 | 100.00 |
|  | 13.48 | 20.37 | 21.89 | 21.35 | 18.21 | 20.04 |
| Hosp. | 15 | 14 | 89 | 140 | 134 | 392 |
|  | 3.83 | 3.57 | 22.70 | 35.71 | 34.18 | 100.00 |
|  | 16.85 | 25.93 | 19.10 | 19.28 | 19.68 | 19.44 |
| Univ. | 16 | 8 | 93 | 139 | 144 | 400 |
|  | 4.00 | 2.00 | 23.25 | 34.75 | 36.00 | 100.00 |
|  | 17.98 | 14.81 | 19.96 | 19.15 | 21.15 | 19.84 |
| Pharm. | 20 | 12 | 89 | 149 | 133 | 403 |
|  | 4.96 | 2.98 | 22.08 | 36.97 | 33.00 | 100.00 |
|  | 22.47 | 22.22 | 19.10 | 20.52 | 19.53 | 19.99 |
| Govt. | 26 | 9 | 93 | 143 | 146 | 417 |
|  | 6.24 | 2.16 | 22.30 | 34.29 | 35.01 | 100.00 |
|  | 29.21 | 16.67 | 19.96 | 19.70 | 21.44 | 20.68 |
| Total | 89 | 54 | 466 | 726 | 681 | 2016 |
|  | 4.41 | 2.68 | 23.12 | 36.01 | 33.78 | 100.00 |
|  | 100.00 | 100.00 | 100.00 | 100.00 | 100.00 | 100.00 |
|  |  |  |  |  |  |  |
|  | Kruskal-Wallis equality-of-populations rank test | | | | |  |
|  | chi-squared = 1.324 with 4 d.f. | | |  |  |  |
|  | probability = 0.8574 | |  |  |  |  |
|  |  |  |  |  |  |  |
|  | chi-squared with ties = 1.467 with 4 d.f. | | | |  |  |
|  | probability = 0.8325 | |  |  |  |  |
|  |  |  |  |  |  |  |

| **Table 2.3** |  |  |  |  |  |  |
| --- | --- | --- | --- | --- | --- | --- |
| Policy: | Individuals will be asked permission for each specific use of their DNA data in the future | | | | | |
|  |  |  |  |  |  |  |
|  | Reduce greatly | Reduce a little | No effect | Increase a little | Increase greatly | Total |
| Tech. | 16 | 21 | 91 | 148 | 128 | 404 |
|  | 3.96 | 5.20 | 22.52 | 36.63 | 31.68 | 100.00 |
|  | 18.18 | 20.59 | 19.91 | 20.76 | 19.51 | 20.04 |
| Hosp. | 18 | 21 | 90 | 133 | 130 | 392 |
|  | 4.59 | 5.36 | 22.96 | 33.93 | 33.16 | 100.00 |
|  | 20.45 | 20.59 | 19.69 | 18.65 | 19.82 | 19.44 |
| Univ. | 15 | 16 | 97 | 138 | 134 | 400 |
|  | 3.75 | 4.00 | 24.25 | 34.50 | 33.50 | 100.00 |
|  | 17.05 | 15.69 | 21.23 | 19.35 | 20.43 | 19.84 |
| Pharm. | 20 | 18 | 84 | 152 | 129 | 403 |
|  | 4.96 | 4.47 | 20.84 | 37.72 | 32.01 | 100.00 |
|  | 22.73 | 17.65 | 18.38 | 21.32 | 19.66 | 19.99 |
| Govt. | 19 | 26 | 95 | 142 | 135 | 417 |
|  | 4.56 | 6.24 | 22.78 | 34.05 | 32.37 | 100.00 |
|  | 21.59 | 25.49 | 20.79 | 19.92 | 20.58 | 20.68 |
| Total | 88 | 102 | 457 | 713 | 656 | 2016 |
|  | 4.37 | 5.06 | 22.67 | 35.37 | 32.54 | 100.00 |
|  | 100.00 | 100.00 | 100.00 | 100.00 | 100.00 | 100.00 |
|  |  |  |  |  |  |  |
|  | Kruskal-Wallis equality-of-populations rank test | | | | |  |
|  | chi-squared = 0.476 with 4 d.f. | | |  |  |  |
|  | probability = 0.9758 | |  |  |  |  |
|  |  |  |  |  |  |  |
|  | chi-squared with ties = 0.524 with 4 d.f. | | | |  |  |
|  | probability = 0.9711 | |  |  |  |  |
|  |  |  |  |  |  |  |

| **Table 2.4** |  |  |  |  |  |  |
| --- | --- | --- | --- | --- | --- | --- |
| Policy: | State-of-the-art IT security are used for all DNA data and other customer data | | | | | |
|  |  |  |  |  |  |  |
|  | Reduce greatly | Reduce a little | No effect | Increase a little | Increase greatly | Total |
| Tech. | 12 | 20 | 149 | 124 | 99 | 404 |
|  | 2.97 | 4.95 | 36.88 | 30.69 | 24.50 | 100.00 |
|  | 13.33 | 21.74 | 20.78 | 19.17 | 21.06 | 20.04 |
| Hosp. | 18 | 21 | 142 | 117 | 94 | 392 |
|  | 4.59 | 5.36 | 36.22 | 29.85 | 23.98 | 100.00 |
|  | 20.00 | 22.83 | 19.80 | 18.08 | 20.00 | 19.44 |
| Univ. | 17 | 18 | 153 | 120 | 92 | 400 |
|  | 4.25 | 4.50 | 38.25 | 30.00 | 23.00 | 100.00 |
|  | 18.89 | 19.57 | 21.34 | 18.55 | 19.57 | 19.84 |
| Pharm. | 21 | 16 | 124 | 149 | 93 | 403 |
|  | 5.21 | 3.97 | 30.77 | 36.97 | 23.08 | 100.00 |
|  | 23.33 | 17.39 | 17.29 | 23.03 | 19.79 | 19.99 |
| Govt. | 22 | 17 | 149 | 137 | 92 | 417 |
|  | 5.28 | 4.08 | 35.73 | 32.85 | 22.06 | 100.00 |
|  | 24.44 | 18.48 | 20.78 | 21.17 | 19.57 | 20.68 |
| Total | 90 | 92 | 717 | 647 | 470 | 2016 |
|  | 4.46 | 4.56 | 35.57 | 32.09 | 23.31 | 100.00 |
|  | 100.00 | 100.00 | 100.00 | 100.00 | 100.00 | 100.00 |
|  |  |  |  |  |  |  |
|  | Kruskal-Wallis equality-of-populations rank test | | | | |  |
|  | chi-squared = 1.811 with 4 d.f. | | |  |  |  |
|  | probability = 0.7706 | |  |  |  |  |
|  |  |  |  |  |  |  |
|  | chi-squared with ties = 1.992 with 4 d.f. | | | |  |  |
|  | probability = 0.7373 | |  |  |  |  |
|  |  |  |  |  |  |  |

| **Table 2.5** |  |  |  |  |  |  |
| --- | --- | --- | --- | --- | --- | --- |
| Policy: | All employees sign an ethical “code of conduct” which includes safeguarding of DNA data | | | | | |
|  |  |  |  |  |  |  |
|  | Reduce greatly | Reduce a little | No effect | Increase a little | Increase greatly | Total |
| Tech. | 20 | 15 | 156 | 122 | 91 | 404 |
|  | 4.95 | 3.71 | 38.61 | 30.20 | 22.52 | 100.00 |
|  | 18.87 | 18.29 | 20.94 | 20.33 | 18.84 | 20.04 |
| Hosp. | 23 | 15 | 151 | 105 | 98 | 392 |
|  | 5.87 | 3.83 | 38.52 | 26.79 | 25.00 | 100.00 |
|  | 21.70 | 18.29 | 20.27 | 17.50 | 20.29 | 19.44 |
| Univ. | 14 | 13 | 148 | 130 | 95 | 400 |
|  | 3.50 | 3.25 | 37.00 | 32.50 | 23.75 | 100.00 |
|  | 13.21 | 15.85 | 19.87 | 21.67 | 19.67 | 19.84 |
| Pharm. | 20 | 13 | 137 | 125 | 108 | 403 |
|  | 4.96 | 3.23 | 34.00 | 31.02 | 26.80 | 100.00 |
|  | 18.87 | 15.85 | 18.39 | 20.83 | 22.36 | 19.99 |
| Govt. | 29 | 26 | 153 | 118 | 91 | 417 |
|  | 6.95 | 6.24 | 36.69 | 28.30 | 21.82 | 100.00 |
|  | 27.36 | 31.71 | 20.54 | 19.67 | 18.84 | 20.68 |
| Total | 106 | 82 | 745 | 600 | 483 | 2016 |
|  | 5.26 | 4.07 | 36.95 | 29.76 | 23.96 | 100.00 |
|  | 100.00 | 100.00 | 100.00 | 100.00 | 100.00 | 100.00 |
|  |  |  |  |  |  |  |
|  | Kruskal-Wallis equality-of-populations rank test | | | | |  |
|  | chi-squared = 7.430 with 4 d.f. | | |  |  |  |
|  | probability = 0.1148 | |  |  |  |  |
|  |  |  |  |  |  |  |
|  | chi-squared with ties = 8.172 with 4 d.f. | | | |  |  |
|  | probability = 0.0855 | |  |  |  |  |
|  |  |  |  |  |  |  |

| **Table 2.6** |  |  |  |  |  |  |
| --- | --- | --- | --- | --- | --- | --- |
| Policy: | Government requests for access to DNA data are refused without a warrant | | | | | |
|  |  |  |  |  |  |  |
|  | Reduce greatly | Reduce a little | No effect | Increase a little | Increase greatly | Total |
| Tech. | 32 | 29 | 128 | 130 | 85 | 404 |
|  | 7.92 | 7.18 | 31.68 | 32.18 | 21.04 | 100.00 |
|  | 17.78 | 24.17 | 20.29 | 20.90 | 18.36 | 20.04 |
| Hosp. | 31 | 22 | 119 | 116 | 104 | 392 |
|  | 7.91 | 5.61 | 30.36 | 29.59 | 26.53 | 100.00 |
|  | 17.22 | 18.33 | 18.86 | 18.65 | 22.46 | 19.44 |
| Univ. | 33 | 21 | 133 | 132 | 81 | 400 |
|  | 8.25 | 5.25 | 33.25 | 33.00 | 20.25 | 100.00 |
|  | 18.33 | 17.50 | 21.08 | 21.22 | 17.49 | 19.84 |
| Pharm. | 37 | 25 | 128 | 115 | 98 | 403 |
|  | 9.18 | 6.20 | 31.76 | 28.54 | 24.32 | 100.00 |
|  | 20.56 | 20.83 | 20.29 | 18.49 | 21.17 | 19.99 |
| Govt. | 47 | 23 | 123 | 129 | 95 | 417 |
|  | 11.27 | 5.52 | 29.50 | 30.94 | 22.78 | 100.00 |
|  | 26.11 | 19.17 | 19.49 | 20.74 | 20.52 | 20.68 |
| Total | 180 | 120 | 631 | 622 | 463 | 2016 |
|  | 8.93 | 5.95 | 31.30 | 30.85 | 22.97 | 100.00 |
|  | 100.00 | 100.00 | 100.00 | 100.00 | 100.00 | 100.00 |
|  |  |  |  |  |  |  |
|  | Kruskal-Wallis equality-of-populations rank test | | | | |  |
|  | chi-squared = 2.467 with 4 d.f. | | |  |  |  |
|  | probability = 0.6505 | |  |  |  |  |
|  |  |  |  |  |  |  |
|  | chi-squared with ties = 2.662 with 4 d.f. | | | |  |  |
|  | probability = 0.6159 | |  |  |  |  |
|  |  |  |  |  |  |  |

| **Table 2.7** |  |  |  |  |  |  |
| --- | --- | --- | --- | --- | --- | --- |
| Policy: | An independent company audits and certifies DNA data security | | | | | |
|  |  |  |  |  |  |  |
|  | Reduce greatly | Reduce a little | No effect | Increase a little | Increase greatly | Total |
| Tech. | 19 | 33 | 170 | 107 | 75 | 404 |
|  | 4.70 | 8.17 | 42.08 | 26.49 | 18.56 | 100.00 |
|  | 14.96 | 23.91 | 22.64 | 17.77 | 18.84 | 20.04 |
| Hosp. | 27 | 22 | 154 | 112 | 77 | 392 |
|  | 6.89 | 5.61 | 39.29 | 28.57 | 19.64 | 100.00 |
|  | 21.26 | 15.94 | 20.51 | 18.60 | 19.35 | 19.44 |
| Univ. | 22 | 23 | 143 | 120 | 92 | 400 |
|  | 5.50 | 5.75 | 35.75 | 30.00 | 23.00 | 100.00 |
|  | 17.32 | 16.67 | 19.04 | 19.93 | 23.12 | 19.84 |
| Pharm. | 25 | 20 | 134 | 147 | 77 | 403 |
|  | 6.20 | 4.96 | 33.25 | 36.48 | 19.11 | 100.00 |
|  | 19.69 | 14.49 | 17.84 | 24.42 | 19.35 | 19.99 |
| Govt. | 34 | 40 | 150 | 116 | 77 | 417 |
|  | 8.15 | 9.59 | 35.97 | 27.82 | 18.47 | 100.00 |
|  | 26.77 | 28.99 | 19.97 | 19.27 | 19.35 | 20.68 |
| Total | 127 | 138 | 751 | 602 | 398 | 2016 |
|  | 6.30 | 6.85 | 37.25 | 29.86 | 19.74 | 100.00 |
|  | 100.00 | 100.00 | 100.00 | 100.00 | 100.00 | 100.00 |
|  |  |  |  |  |  |  |
|  | Kruskal-Wallis equality-of-populations rank test | | | | |  |
|  | chi-squared = 10.037 with 4 d.f. | | |  |  |  |
|  | probability = 0.0398 | |  |  |  |  |
|  |  |  |  |  |  |  |
|  | chi-squared with ties = 10.989 with 4 d.f. | | | |  |  |
|  | probability = 0.0267 | |  |  |  |  |
|  |  |  |  |  |  |  |

| **Table 2.8** |  |  |  |  |  |  |
| --- | --- | --- | --- | --- | --- | --- |
| Policy: | Access to family medical records is required, and these will be linked to DNA data | | | | | |
|  |  |  |  |  |  |  |
|  | Reduce greatly | Reduce a little | No effect | Increase a little | Increase greatly | Total |
| Tech. | 71 | 79 | 141 | 79 | 34 | 404 |
|  | 17.57 | 19.55 | 34.90 | 19.55 | 8.42 | 100.00 |
|  | 23.13 | 22.38 | 19.53 | 20.36 | 13.82 | 20.04 |
| Hosp. | 49 | 83 | 140 | 72 | 48 | 392 |
|  | 12.50 | 21.17 | 35.71 | 18.37 | 12.24 | 100.00 |
|  | 15.96 | 23.51 | 19.39 | 18.56 | 19.51 | 19.44 |
| Univ. | 58 | 59 | 161 | 77 | 45 | 400 |
|  | 14.50 | 14.75 | 40.25 | 19.25 | 11.25 | 100.00 |
|  | 18.89 | 16.71 | 22.30 | 19.85 | 18.29 | 19.84 |
| Pharm. | 53 | 68 | 137 | 87 | 58 | 403 |
|  | 13.15 | 16.87 | 34.00 | 21.59 | 14.39 | 100.00 |
|  | 17.26 | 19.26 | 18.98 | 22.42 | 23.58 | 19.99 |
| Govt. | 76 | 64 | 143 | 73 | 61 | 417 |
|  | 18.23 | 15.35 | 34.29 | 17.51 | 14.63 | 100.00 |
|  | 24.76 | 18.13 | 19.81 | 18.81 | 24.80 | 20.68 |
| Total | 307 | 353 | 722 | 388 | 246 | 2016 |
|  | 15.23 | 17.51 | 35.81 | 19.25 | 12.20 | 100.00 |
|  | 100.00 | 100.00 | 100.00 | 100.00 | 100.00 | 100.00 |
|  |  |  |  |  |  |  |
|  | Kruskal-Wallis equality-of-populations rank test | | | | |  |
|  | chi-squared = 8.293 with 4 d.f. | | |  |  |  |
|  | probability = 0.0814 | |  |  |  |  |
|  |  |  |  |  |  |  |
|  | chi-squared with ties = 8.858 with 4 d.f. | | | |  |  |
|  | probability = 0.0647 | |  |  |  |  |
|  |  |  |  |  |  |  |

| **Table 2.9** |  |  |  |  |  |  |
| --- | --- | --- | --- | --- | --- | --- |
| Policy: | Members of the general public serve on a committee to decide how genomic data will be used | | | | | |
|  |  |  |  |  |  |  |
|  | Reduce greatly | Reduce a little | No effect | Increase a little | Increase greatly | Total |
| Tech. | 57 | 55 | 169 | 81 | 42 | 404 |
|  | 14.11 | 13.61 | 41.83 | 20.05 | 10.40 | 100.00 |
|  | 20.36 | 18.21 | 19.07 | 21.66 | 24.14 | 20.04 |
| Hosp. | 51 | 61 | 176 | 70 | 34 | 392 |
|  | 13.01 | 15.56 | 44.90 | 17.86 | 8.67 | 100.00 |
|  | 18.21 | 20.20 | 19.86 | 18.72 | 19.54 | 19.44 |
| Univ. | 44 | 64 | 177 | 83 | 32 | 400 |
|  | 11.00 | 16.00 | 44.25 | 20.75 | 8.00 | 100.00 |
|  | 15.71 | 21.19 | 19.98 | 22.19 | 18.39 | 19.84 |
| Pharm. | 58 | 58 | 177 | 74 | 36 | 403 |
|  | 14.39 | 14.39 | 43.92 | 18.36 | 8.93 | 100.00 |
|  | 20.71 | 19.21 | 19.98 | 19.79 | 20.69 | 19.99 |
| Govt. | 70 | 64 | 187 | 66 | 30 | 417 |
|  | 16.79 | 15.35 | 44.84 | 15.83 | 7.19 | 100.00 |
|  | 25.00 | 21.19 | 21.11 | 17.65 | 17.24 | 20.68 |
| Total | 280 | 302 | 886 | 374 | 174 | 2016 |
|  | 13.89 | 14.98 | 43.95 | 18.55 | 8.63 | 100.00 |
|  | 100.00 | 100.00 | 100.00 | 100.00 | 100.00 | 100.00 |
|  |  |  |  |  |  |  |
|  | Kruskal-Wallis equality-of-populations rank test | | | | |  |
|  | chi-squared = 6.228 with 4 d.f. | | |  |  |  |
|  | probability = 0.1828 | |  |  |  |  |
|  |  |  |  |  |  |  |
|  | chi-squared with ties = 6.904 with 4 d.f. | | | |  |  |
|  | probability = 0.1410 | |  |  |  |  |
|  |  |  |  |  |  |  |

| **Table 2.10** |  |  |  |  |  |  |
| --- | --- | --- | --- | --- | --- | --- |
| Policy: | Individuals’ DNA data are stored indefinitely | | | |  |  |
|  |  |  |  |  |  |  |
|  | Reduce greatly | Reduce a little | No effect | Increase a little | Increase greatly | Total |
| Tech. | 65 | 53 | 194 | 49 | 43 | 404 |
|  | 16.09 | 13.12 | 48.02 | 12.13 | 10.64 | 100.00 |
|  | 22.34 | 16.11 | 20.84 | 18.01 | 22.28 | 20.04 |
| Hosp. | 51 | 61 | 194 | 48 | 38 | 392 |
|  | 13.01 | 15.56 | 49.49 | 12.24 | 9.69 | 100.00 |
|  | 17.53 | 18.54 | 20.84 | 17.65 | 19.69 | 19.44 |
| Univ. | 46 | 71 | 195 | 61 | 27 | 400 |
|  | 11.50 | 17.75 | 48.75 | 15.25 | 6.75 | 100.00 |
|  | 15.81 | 21.58 | 20.95 | 22.43 | 13.99 | 19.84 |
| Pharm. | 65 | 67 | 165 | 61 | 45 | 403 |
|  | 16.13 | 16.63 | 40.94 | 15.14 | 11.17 | 100.00 |
|  | 22.34 | 20.36 | 17.72 | 22.43 | 23.32 | 19.99 |
| Govt. | 64 | 77 | 183 | 53 | 40 | 417 |
|  | 15.35 | 18.47 | 43.88 | 12.71 | 9.59 | 100.00 |
|  | 21.99 | 23.40 | 19.66 | 19.49 | 20.73 | 20.68 |
| Total | 291 | 329 | 931 | 272 | 193 | 2016 |
|  | 14.43 | 16.32 | 46.18 | 13.49 | 9.57 | 100.00 |
|  | 100.00 | 100.00 | 100.00 | 100.00 | 100.00 | 100.00 |
|  |  |  |  |  |  |  |
|  | Kruskal-Wallis equality-of-populations rank test | | | | |  |
|  | chi-squared = 1.136 with 4 d.f. | | |  |  |  |
|  | probability = 0.8885 | |  |  |  |  |
|  |  |  |  |  |  |  |
|  | chi-squared with ties = 1.276 with 4 d.f. | | | |  |  |
|  | probability = 0.8655 | |  |  |  |  |
|  |  |  |  |  |  |  |

| **Table 2.11** |  |  |  |  |  |  |
| --- | --- | --- | --- | --- | --- | --- |
| Policy: | Copies of all DNA data (without individuals’ names) are deposited into a government database | | | | | |
|  |  |  |  |  |  |  |
|  | Reduce greatly | Reduce a little | No effect | Increase a little | Increase greatly | Total |
| Tech. | 87 | 74 | 155 | 54 | 34 | 404 |
|  | 21.53 | 18.32 | 38.37 | 13.37 | 8.42 | 100.00 |
|  | 20.67 | 19.53 | 20.58 | 19.78 | 17.89 | 20.04 |
| Hosp. | 80 | 75 | 149 | 50 | 38 | 392 |
|  | 20.41 | 19.13 | 38.01 | 12.76 | 9.69 | 100.00 |
|  | 19.00 | 19.79 | 19.79 | 18.32 | 20.00 | 19.44 |
| Univ. | 89 | 83 | 145 | 52 | 31 | 400 |
|  | 22.25 | 20.75 | 36.25 | 13.00 | 7.75 | 100.00 |
|  | 21.14 | 21.90 | 19.26 | 19.05 | 16.32 | 19.84 |
| Pharm. | 94 | 72 | 139 | 53 | 45 | 403 |
|  | 23.33 | 17.87 | 34.49 | 13.15 | 11.17 | 100.00 |
|  | 22.33 | 19.00 | 18.46 | 19.41 | 23.68 | 19.99 |
| Govt. | 71 | 75 | 165 | 64 | 42 | 417 |
|  | 17.03 | 17.99 | 39.57 | 15.35 | 10.07 | 100.00 |
|  | 16.86 | 19.79 | 21.91 | 23.44 | 22.11 | 20.68 |
| Total | 421 | 379 | 753 | 273 | 190 | 2016 |
|  | 20.88 | 18.80 | 37.35 | 13.54 | 9.42 | 100.00 |
|  | 100.00 | 100.00 | 100.00 | 100.00 | 100.00 | 100.00 |
|  |  |  |  |  |  |  |
|  | Kruskal-Wallis equality-of-populations rank test | | | | |  |
|  | chi-squared = 5.928 with 4 d.f. | | |  |  |  |
|  | probability = 0.2046 | |  |  |  |  |
|  |  |  |  |  |  |  |
|  | chi-squared with ties = 6.383 with 4 d.f. | | | |  |  |
|  | probability = 0.1723 | |  |  |  |  |
|  |  |  |  |  |  |  |

| **Table 2.12** |  |  |  |  |  |  |
| --- | --- | --- | --- | --- | --- | --- |
| Policy: | Access to DNA data is sold to pharmaceutical firms (without requesting further permission) | | | | | |
|  |  |  |  |  |  |  |
|  | Reduce greatly | Reduce a little | No effect | Increase a little | Increase greatly | Total |
| Tech. | 155 | 112 | 93 | 24 | 20 | 404 |
|  | 38.37 | 27.72 | 23.02 | 5.94 | 4.95 | 100.00 |
|  | 17.65 | 23.53 | 21.88 | 18.32 | 18.87 | 20.04 |
| Hosp. | 175 | 83 | 94 | 22 | 18 | 392 |
|  | 44.64 | 21.17 | 23.98 | 5.61 | 4.59 | 100.00 |
|  | 19.93 | 17.44 | 22.12 | 16.79 | 16.98 | 19.44 |
| Univ. | 177 | 99 | 85 | 24 | 15 | 400 |
|  | 44.25 | 24.75 | 21.25 | 6.00 | 3.75 | 100.00 |
|  | 20.16 | 20.80 | 20.00 | 18.32 | 14.15 | 19.84 |
| Pharm. | 177 | 105 | 68 | 27 | 26 | 403 |
|  | 43.92 | 26.05 | 16.87 | 6.70 | 6.45 | 100.00 |
|  | 20.16 | 22.06 | 16.00 | 20.61 | 24.53 | 19.99 |
| Govt. | 194 | 77 | 85 | 34 | 27 | 417 |
|  | 46.52 | 18.47 | 20.38 | 8.15 | 6.47 | 100.00 |
|  | 22.10 | 16.18 | 20.00 | 25.95 | 25.47 | 20.68 |
| Total | 878 | 476 | 425 | 131 | 106 | 2016 |
|  | 43.55 | 23.61 | 21.08 | 6.50 | 5.26 | 100.00 |
|  | 100.00 | 100.00 | 100.00 | 100.00 | 100.00 | 100.00 |
|  |  |  |  |  |  |  |
|  | Kruskal-Wallis equality-of-populations rank test | | | | |  |
|  | chi-squared = 2.302 with 4 d.f. | | |  |  |  |
|  | probability = 0.6804 | |  |  |  |  |
|  |  |  |  |  |  |  |
|  | chi-squared with ties = 2.574 with 4 d.f. | | | |  |  |
|  | probability = 0.6315 | |  |  |  |  |
